# Micro-CT yields high image quality in human fetal post-mortem imaging despite maceration

**DOI:** 10.1186/s12880-021-00658-5

**Published:** 2021-08-24

**Authors:** Ian Craig Simcock, Susan Cheng Shelmerdine, Dean Langan, Guy Anna, Neil James Sebire, Owen John Arthurs

**Affiliations:** 1 Department of Clinical Radiology, Great Ormond Street Hospital for Children, London, UK; 2 Great Ormond Street Hospital for Children, UCL Great Ormond Street Institute of Child Health, London, UK; 3 NIHR Great Ormond Street Hospital Biomedical Research Centre, London, UK; 4 Department of Histopathology, Great Ormond Street Hospital for Children, London, UK

**Keywords:** Micro-CT, Human foetuses, Post-mortem imaging, Maceration, Image quality

## Abstract

**Background:**

Current clinical post-mortem imaging techniques do not provide sufficiently high-resolution imaging for smaller fetuses after pregnancy loss. Post-mortem micro-CT is a non-invasive technique that can deliver high diagnostic accuracy for these smaller fetuses. The purpose of the study is to identify the main predictors of image quality for human fetal post-mortem micro-CT imaging.

**Methods:**

Human fetuses were imaged using micro-CT following potassium tri-iodide tissue preparation, and axial head and chest views were assessed for image quality on a Likert scale by two blinded radiologists. Simple and multivariable linear regression models were performed with demographic details, iodination, tissue maceration score and imaging parameters as predictor variables.

**Results:**

258 fetuses were assessed, with median weight 41.7 g (2.6–350 g) and mean gestational age 16 weeks (11–24 weeks). A high image quality score (> 6.5) was achieved in 95% of micro-CT studies, higher for the head (median = 9) than chest (median = 8.5) imaging. The strongest negative predictors of image quality were increasing maceration and body weight (*p* < 0.001), with number of projections being the best positive imaging predictor.

**Conclusions:**

High micro-CT image quality score is achievable following early pregnancy loss despite fetal maceration, particularly in smaller fetuses where conventional autopsy may be particularly challenging. These findings will help establish clinical micro-CT imaging services, addressing the need for less invasive fetal autopsy methods.

## Background

Conventional perinatal autopsy can determine a cause of death or diagnosis by identifying developmental abnormalities and reducing the discrepancy between the ante- and post-mortem diagnosis [1]. However, parental uptake for conventional autopsy has been in decline for several decades due to its invasive nature [2, 3]. Minimally-invasive imaging techniques which are more acceptable to parents [3–5] have been developed to determine the causes of miscarriage and stillbirth, and to assist in the planning of future pregnancies [6–10]. Most current clinically available scanning methods do not provide sufficient image resolution of smaller fetuses [9, 11], necessitating trialling higher resolution techniques [10, 12–14].

Micro-CT is a technique that can achieve high resolution of anatomical structures [15–19] and with the addition of a potassium tri-iodide (I_2_KI) contrast agent can provide detailed soft tissue imaging which can identify developmental abnormalities in early pregnancy where conventional autopsy is challenging [15, 16, 20–24]. Micro CT has been used to image human fetuses with high diagnostic accuracy [20, 25], but the optimal imaging parameters across a range of fetal size, gestation, and maceration and how these relate to image quality have yet to be determined. The aim of this study was to identify the strongest demographic or imaging-derived determinants of fetal post-mortem micro-CT imaging.

## Methods

Ethical approval was granted for this retrospective, single centre study (13/LO/1994 and 17/WS/0089) with all specimens being handled according to the Human Tissue Act (2004). Parental consent was obtained for post-mortem imaging.

### Patient selection

Consecutive unselected fetuses under 350 g body weight from a 3-year period (January 2017 to November 2019) underwent whole body micro-CT imaging as part of the consented autopsy examination for clinical care. Prior to imaging, all fetuses were stored in the hospital mortuary and refrigerated at 4 °C. Fetuses were immersed in a solution of 2.5% I_2_KI (2–13 days) to allow full iodination through diffusion of the contrast to the centre of the fetus according to established protocols [26]. We used 5 days per 100 g body weight as an estimation of time to iodination.

### Post-mortem micro-CT imaging

Micro-CT imaging was completed using one of two Nikon micro-CT scanners depending on machine availability (model: Med-X or XTH 225-ST; Nikon, Tring, UK). Two micro-CT studies were performed for each fetus to allow for highest resolution and magnification imaging as possible: a dedicated head study, and combined chest abdomen pelvis study [20]. The micro-CT imaging was completed by one of three operators, each with at least 4 years of scanning experience.

Fetuses were secured within the scanner using foam supports, moisture absorbent wrapping material and Parafilm M (Bemis, Oshkosh, USA) to ensure mechanical stability [27]. Projection images acquired by the scanner were reconstructed using modified Feldkamp filtered back-projection algorithms with proprietary software (CTPro3D; Nikon Metrology, UK) and post processed using VGStudio MAX 3.4 (Volume Graphics GmbH, Heidelberg, Germany). Isotropic voxel sizes varied according to specimen size and magnification, ranging from 11.6 to 89.3 µm. We applied beam hardening correction, no noise reduction median filter or tube filtration.

### Demographic data and imaging factors

Demographical data included gestational age (weeks), post-mortem weight (g), post-mortem interval (days), mode of death, and three fetal measurements in centimetres (crown-rump length (CRL), crown-heel length (CHL) and head circumference (HC) as well as whether an abnormality was detected by micro-CT (yes/no).

The timings between delivery and imaging were recorded, and comprised of:

1. Time to immersion (defined as the time from birth to placement in I_2_KI solution) and
2. Iodination time (defined as the time spent in I_2_KI solution prior to micro-CT imaging).

Specialist paediatric pathologists subjectively assessed the degree of maceration at external examination for the whole body, and derived a single score for each fetus, based on previously published work (0 = no maceration, 3 severe/established maceratio[28, 29]).

Imaging parameters were recorded for the head and chest/abdomen/pelvis examinations and fell within the following ranges: kilovoltage (60–160 kilovolts), current (78–400 µA), power (7–40 Watts), exposure time (125–1000 ms), frames per projection (1–4) projections (1066–3141) and effective pixel size (EPS) (11.6–89.3 microns).

### Image analysis

Image quality assessments were completed independently by two board-certified paediatric radiologists, with 5 years (SS) and 16 years (OA) of specialist post-mortem radiology experience. All images were anonymised, and observers were blinded to the clinical history and pathological assessments.

Image quality assessments were made on 2 selected axial images per fetus, one through the head at the level of the mid-brain, and one through the thorax at the level of the heart, providing a 4-chamber cardiac view.

**Fig. 1:**
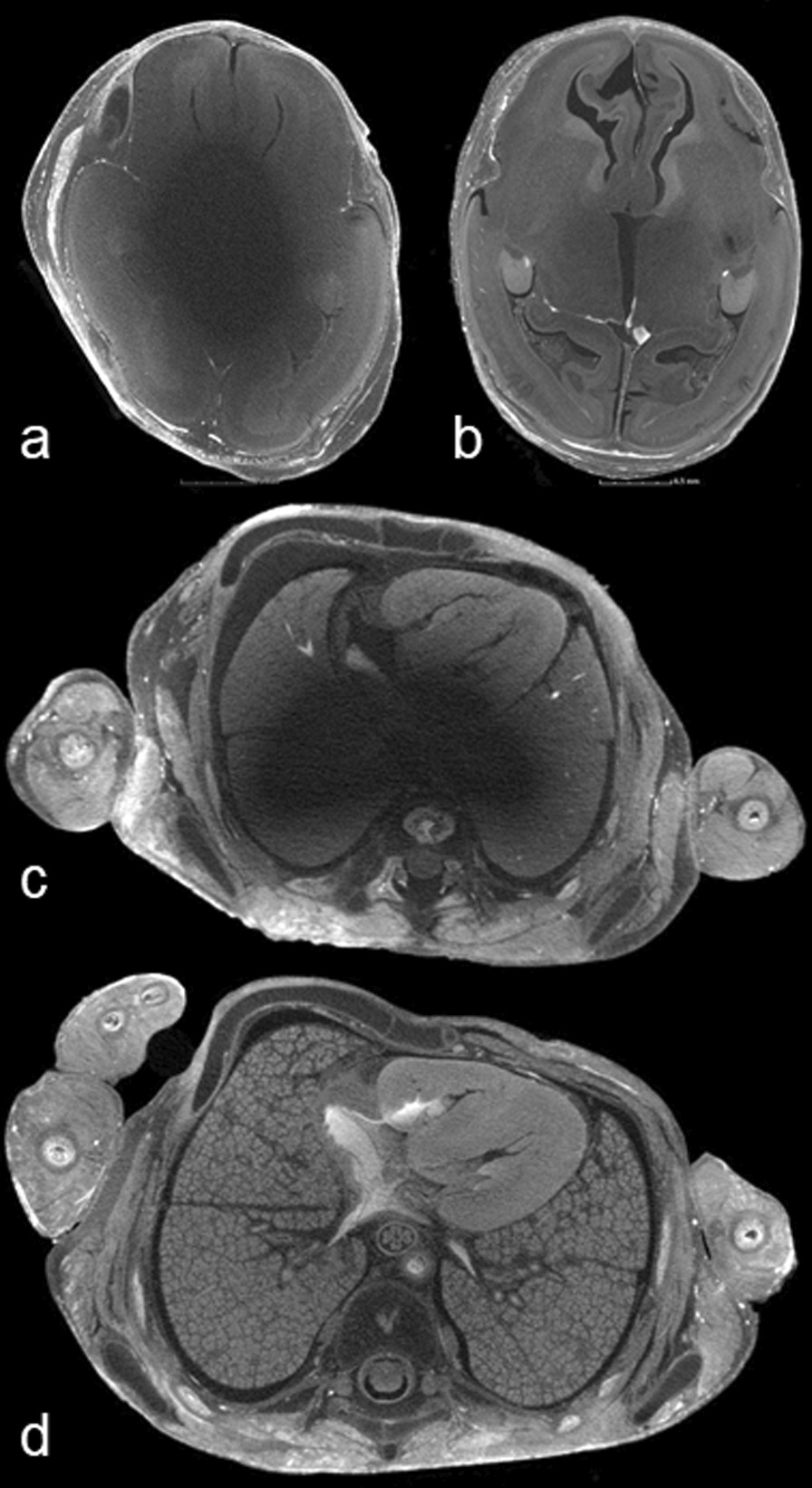
Axial micro-CT images acquired through the head (**a,b**) and chest (**c,d**) demonstrating incomplete (**a, c**) and complete (**b, d**) iodination. The areas of lower density (i.e., black) seen within the central portion of the images (**a, c**) demonstrate incomplete iodine penetration through the body, and hamper diagnostic interpretation of soft tissue structures. (Axial micro-CT images acquired at 100kv, 150uA, 354 ms, 1 frames per projection) (fpp) and 3141 number of projections.)

**Fig. 2:**
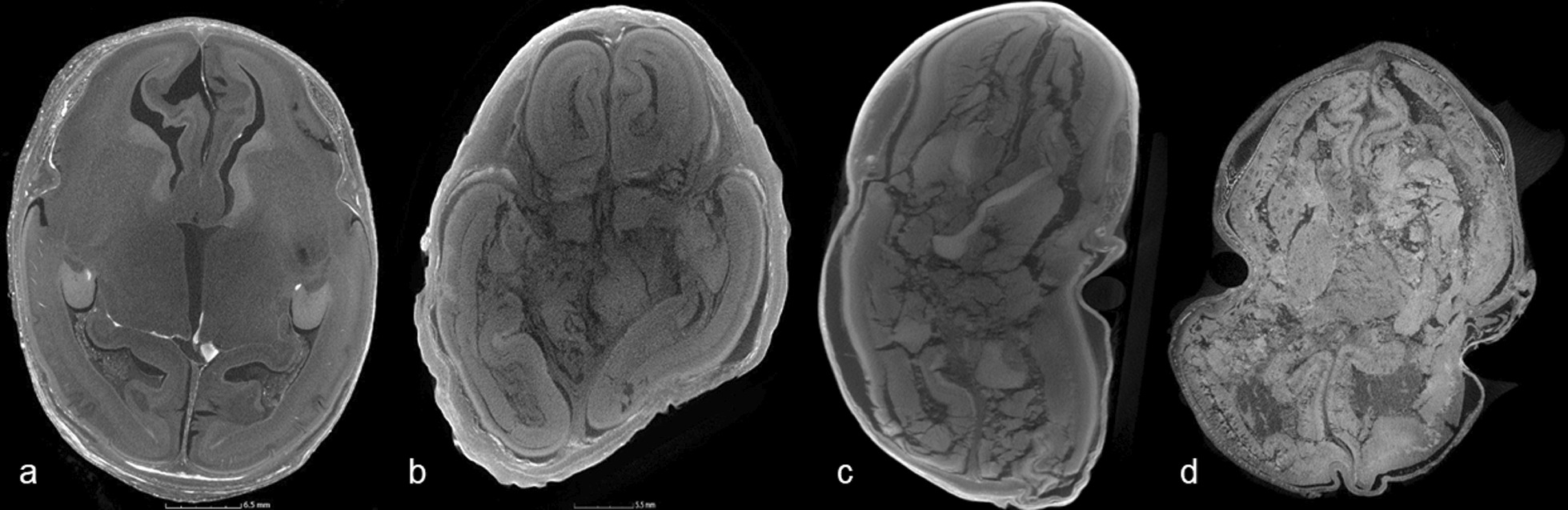
Axial micro-CT head images of four different fetuses demonstrating different degrees of maceration. **a** No maceration, **b** mild cracking and distortion of the image (add arrows), **c** moderate disruption with reduction in tissue planes (arrow), **d** non-diagnostic severe maceration

Each radiologist provided three different subjective image quality assessments:

1. Full iodination (yes, diagnostic/no, non-diagnostic) Fig. 1.
2. Imaging maceration score

(Based on a scale 0–3: where 0 = none, 1 = mild cracking, distortion but images diagnostic, 2 = moderate disruption with reduction in normal tissue planes, limited diagnosis, 3 = severe maceration, non-diagnostic with severely distorted internal anatomy) Fig. 2, and

**Fig. 3:**
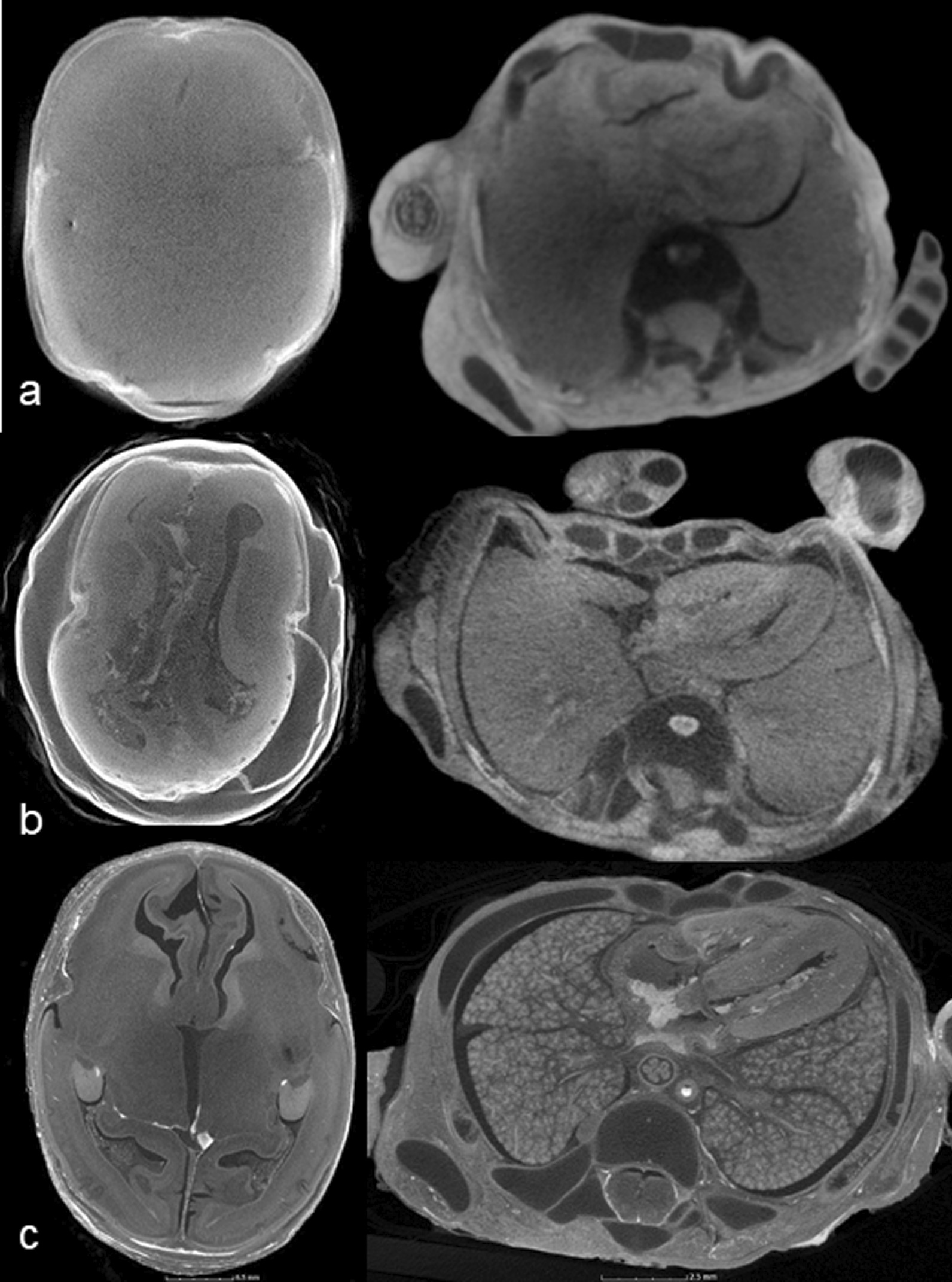
Axial micro-CT images through the head and chest in different fetuses, demonstrating differences in image quality. Images which were grainy and tissue planes indistinct were assessed as poor quality (**a**), with residual image graininess in moderate quality (**b**), which was imperceptible in high quality (**c**)

3. Image quality score

(Based on a scale from 0 to 10: where 0–3 = poor image quality, very grainy images, loss of normal tissue planes; 4–6 = moderate image quality, some graininess perceptible, but major structures and tissue planes unaffected; 7–10 = high image quality, imperceptible graininess in image, excellent detail), Fig. 3. This score does not take into account iodination or maceration score.

### Statistical analysis

Descriptive statistics were calculated for baseline demographics, tissue preparation variables and image parameters. Inter- and intra-rater reliability was investigated through the kappa statistic (with linear weights) for maceration score and summary statistics (median, IQR) presented for the differences between image quality ratings. The average ratings between the two radiologist readers for image quality (the primary outcome) and maceration score were used as the final outcome for subsequent analyses.

Simple and multivariable linear regression models were fitted for the outcome variables of image quality and perceived maceration score, separately for head and chest images. Baseline demographics, tissue preparation variables and image parameters were considered as predictor variables in these models. A forward and backward model selection approach was adopted to minimise the model fit statistic AIC to choose the final multivariable models. All analyses were carried out in R version 3.6.1.

## Results

### Demographics and imaging parameters

**Fig. 4:**
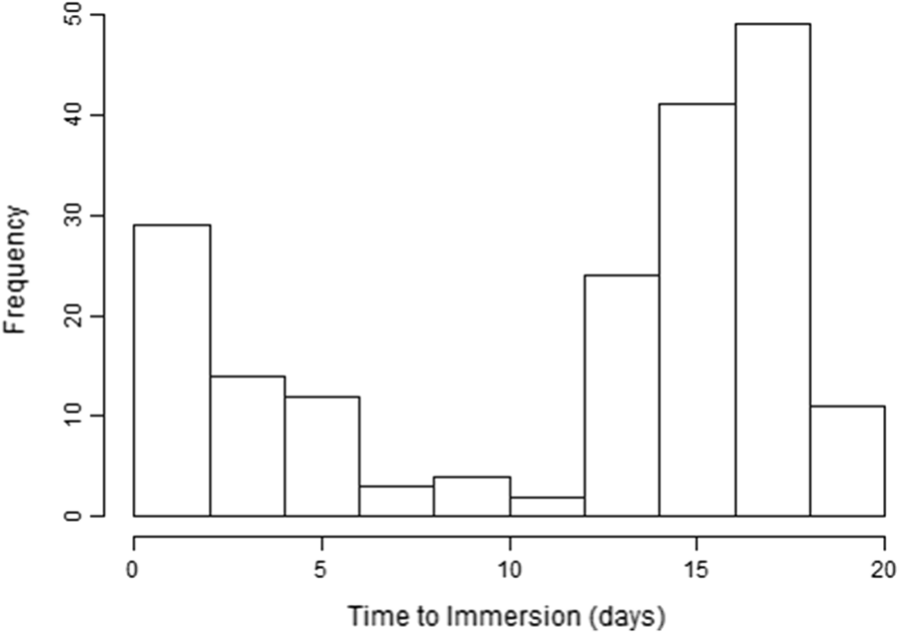
Bimodal distribution of time to immersion across the fetuses included in our cohort

258 fetuses underwent micro-CT with a median post-mortem weight 41.7 g (range 2.6–350), mean gestational age of 16 weeks (range 11–24). Further demographic data is provided in Table 1. The time to immersion showed a bimodal distribution with peaks < 10 days (median 3) and > 10 days (median 16 days), Fig. 4.

**Table 1:** Study cohort demographic data and imaging parameter range

	N	Min	Max	Mean	SD	Median	Lower Qu	Upper Qu
*Patient Baseline demographics*								
Gestational age (weeks)	258	11	24	16.0	2.50	16.0	14.0	17.8
Post mortem interval (days)	258	0	48	14.1	5.21	13	11.0	17.0
Crown rump length (cm)	258	4.0	18.6	10.4	2.92	10	8.0	12.5
Crown heel length (cm) (missing = 1)	257	5.7	26.2	14.3	4.25	14.1	11.0	17.4
Head circumference (cm)	258	4.1	16.9	9.60	2.91	9.3	7.2	11.9
Post mortem weight (g)	258	2.6	350.0	64.8	63.4	41.7	20.5	94.1
*Tissue preparation*								
Time to immersion (missing = 69)								
≤ 10 days	62	1	10	3.48	2.49	3	1	5
> 10 days	127	11	19	16.2	1.80	16	15	18
Time Iodinated (days) (missing = 69)	189	1	14	8.80	2.55	9.0	8.0	10.0
*Imaging parameters*								
Kilovoltage	258	60	160	106	14.7	100	100	120
Current (µA)	258	78	400	142	44.6	130	120	150
Power (Watts)	258	7	40	15	4.9	14	12	17
Projections	258	1351	3141	2716	440	2808	2431	3141

Approximately a quarter (68/258, 26.5%) of cases showed significant abnormality on micro-CT, the results of which have been presented elsewhere [20]. The imaging indication was mostly miscarriage 157/258 (60.9%), followed by termination of pregnancy (TOP) 69/258 (26.7%) and intrauterine death (IUD) 32/258 (12.4%). The range of imaging parameters used were the same for head and chest imaging (Table 1) with minor differences between examination areas in < 5% of cases (kilovoltage (kV), current, exposure time, power), apart from number of projections and estimated pixel size which varied to a greater degree due to anatomical area size (Table 1).

### Overall image quality assessments

**Fig. 5:**
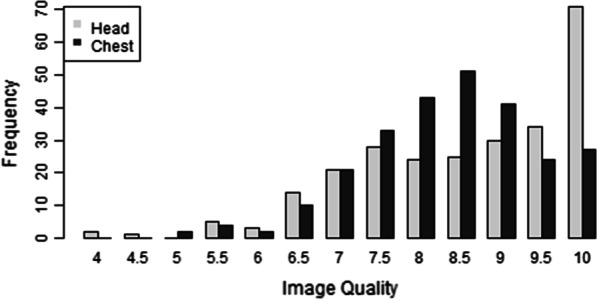
Difference in range in image quality scores between head and chest axial images, with head displaying greater overall image quality than chest.”

An image quality score of greater than 6.5 was recorded in 247/258 (95.8%) head and 251/258 (97.3%) chest imaging (i.e., 497/516 (95.9%) images overall). Image quality scores were higher for head (median = 9, range 4–10) than for chest (median = 8.5, range 5–10; *p* < 0.001; Fig. 5).

Complete agreement between the two observers was seen in 51.2% (132/258) head images and 36% (93/258) chest images with mean score used as “image quality” for the results.

### Maceration and iodination assessments

250/258 (96.9%) of all cases were classed as fully iodinated. There was high agreement of iodination status for heads 250/258 (96.8%) and chest 252/258 (97.6%).

Imaging of the brain scored greater for maceration than the chest. In just over a quarter of brain imaging cases (69/258, 26.7%) the highest maceration imaging score of 3 was provided, compared to only 3/258 (1.2%) chest images. There was an acceptable inter-rater agreement for the maceration score, with weighted Kappa = 0.56 (95% CI 0.49–0.63, *p* < 0.001).

Pathologists at external examination however recorded that over half of all fetuses 133/258, 51.6%) were severely macerated (i.e., score 3); a minority (14/258 (5.4%) as moderately macerated (i.e., score 2) or mildly macerate (i.e., score 1, 30 / 258, 11.6%) and approximately a third (81/258, 31.4%) as not macerated.

There was agreement of maceration score on micro-CT images and pathological assessment (within ± 0.5) for the head in 150/258 (58.1%), and chest in 107/258 (41.5%), with micro-CT images predominantly scoring less severe maceration in (64/258) 24.8% head and 138/258 (53.5%) chest imaging than external assessment.

### Image quality predictors

The main predictor of image quality for both head and chest was the pathological score of maceration, with a more severe maceration score associated with lower image quality (*p* < 0.001), although this only accounted for 6.6% of the variation for head imaging and 11.6% for chest imaging. Increasing gestational weight was also negatively associated with image quality in the adjusted models (*p* < 0.001 head, *p* = 0.003 chest), but not in unadjusted models.

The main imaging parameter predictor of the image quality score was the number of projections acquired, which was positively associated with image quality in both head and chest models. A greater image quality score was also positively associated with frames per projection (for 2 frames, as opposed to 1 or 4 frames) in both head (*p* < 0.081) and chest (*p* < 0.001) images.

Time to immersion was also negatively associated with image quality in chest images with borderline significance (*p* = 0.039), although there were several missing datapoints (69/258; 26.7%) where this was not recorded in the clinical notes. EPS was also negatively associated (Table 2).

**Table 2:** Simple and multivariable linear regression analysis

	Head images							Chest images						
	Unadjusted				Adjusted			Unadjusted				Adjusted		
	OR	95% CI	*p*-value	R^2^ (%)	OR	95% CI	*p*-value	OR	95% CI	*p*-value	R^2^	OR	95% CI	*p*-value
Patient demographics														
Gestational Age (weeks)	− 0.014	− 0.079, 0.052	0.678	0.1	0.076	− 0.000, 0.152	0.050	− 0.062	− 0.113, − 0.010	0.020	2.1	–	–	–
Post Mortem Interval (days)	− 0.008	− 0.039, 0.024	0.638	0.1	–	–	–	− 0.005	− 0.030, 0.020	0.685	0.1	–	–	–
Crown Rump Length (cm)	0.004	− 0.052, 0.060	0.891	0.0	–	–	–	− 0.034	− 0.078, 0.011	0.138	0.9	–	–	–
Crown Heel Length (cm)	0.012	− 0.027, 0.050	0.549	0.1	–	–	–	− 0.018	− 0.049, 0.013	0.254	0.5	–	–	–
Head Circumference (cm)	0.021	− 0.035, 0.077	0.464	0.2	–	–	–	− 0.005	− 0.050, 0.040	0.822	0.0	–	–	–
Post Mortem Weight (100 g)	− 0.004	− 0.263, 0.255	0.976	0.0	− 0.716	− 1.074, − 0.357	< 0.001	− 0.029	− 0.235, 0.178	0.785	0.0	− 0.337	− 0.550, − 0.114	0.003
*Ab on Micro-CT (ref = no)*														
Yes	0.232	− 0.139, 0.602	0.220	0.6	–	–	–	0.367	0.073, 0.660	0.015	2.6	–	–	–
*Mode of death* (ref = IUD)														
Miscarriage	0.619	0.126, 1.110	0.014		0.469	− 0.003, 0.940	0.051	0.219	− 0.170, 0.608	0.269		− 0.112	− 0.480, 0.256	0.548
TOP	1.170	0.623, 1.710	< 0.001	6.9	0.826	0.253, 1.400	0.005	0.895	0.466, 1.320	< 0.001	9.3	0.326	− 0.122, 0.775	0.152
Maceration score (autopsy, ref = 0)														
1	− 0.410	− 0.956, 0.136	0.140		− 0.523	− 1.027, − 0.020	0.042	− 0.450	− 0.874, − 0.026	0.038		− 0.616	− 1.003, − 0.229	0.001
2	− 1.110	− 1.85, − 0.376	0.003		− 0.931	− 1.652, − 0.211	0.012	− 0.655	− 1.230, − 0.081	0.026		− 0.815	− 1.370, − 0.260	0.004
3	− 0.690	− 1.050, − 0.330	< 0.001	6.6	− 0.592	− 1.048, − 0.136	0.011	− 0.815	− 1.090, − 0.535	< 0.001	11.6	− 0.830	− 1.158, − 0.500	< 0.001
*Tissue Preparation*														
Time to immersion (days)	− 0.006	− 0.037, 0.024	0.694	0.1	–	–	–	− 0.024	− 0.046, − 0.001	0.039	2.3	–	–	–
Time iodinated (days)	0.036	− 0.040, 0.111	0.357	0.5	–	–	–	0.038	− 0.018, 0.094	0.186	0.9	–	–	–
*Imaging parameters*														
Kilovoltage (per 100)	− 0.095	− 1.210, 1.020	0.867	0.0	–	–	–	0.373	− 0.516, 1.260	0.410	0.3	–	–	–
Current (100 µA)	0.060	− 0.295, 0.415	0.740	0.0	–	–	–	− 0.170	− 0.463, 0.123	0.254	0.5	–	–	–
Power (10 W)	0.054	− 0.273, 0.381	0.744	0.0	–	–	–	− 0.099	− 0.367, 0.169	0.469	0.2	− 0.289	− 0.548, − 0.029	0.029
Projections (100 s)	0.095	0.065, 0.126	< 0.001	12.8	0.102	0.070, 0.134	< 0.001	0.060	0.031, 0.088	< 0.001	6.0	0.044	0.015, 0.072	0.002
EPS (100 microns)	− 2.150	− 3.310, − 0.982	< 0.001	4.9	–	–	–	− 1.650	− 2.590, − 0.701	< 0.001	4.4	–	–	–
Exposure time (100 ms)	0.048	− 0.075, 0.170	0.445	0.2	–	–	–	0.071	− 0.027, 0.168	0.153	0.8	0.074	− 0.022, 0.170	0.132
Frames per projection (ref = 1)														
2	0.332	− 0.041, 0.706	0.081		–	–	–	0.600	0.291, 0.909	< 0.001		0.639	0.341, 0.937	< 0.001
4	− 0.093	− 1.210, 1.020	0.869	1.3	–	–	–	− 0.453	− 1.270, 0.360	0.273	7.3	0.178	− 0.566, 0.921	0.638
*Target (ref = Mo)*														
W	0.165	− 0.383, 0.713	0.554	0.1	–	–	–	0.447	0.015, 0.879	0.043	1.6	0.358	− 0.058, 0.774	0.091

Table 2 Simple and multivariable linear regression. Maceration was the main predictor of image quality, with poorer overall image quality at higher maceration scores. Higher gestational weight was also negatively associated with image quality in the adjusted models. Time to immersion was also negatively associated with image quality for chest images. The number of projections was the main imaging parameter associated with higher image quality scores

OR: Odds ratio, CI: confidence interval

## Discussion

In this study, a high image quality score (of over 6.5) was achieved in the clear majority (96%) of fetal head and chest micro-CT studies from a range of gestational weights. The strongest negative predictor of final image quality was the extent of fetal maceration, with decreasing image quality also associated with increasing body weight. The strongest imaging parameter predictors for image quality were the number of frames per projection and EPS.

There was some discrepancy between micro-CT imaging assessment and pathological visual assessment of the extent of fetal maceration, where pathological maceration assessment was a better predictor of poor micro-CT image quality, although poor image quality was only found in 4.1% of fetuses overall. This means that we achieved good micro-CT image quality in the overwhelming majority of cases despite several severely macerated fetuses being referred to our unit, which will help the more widespread use of high-resolution imaging in macerated cases. Therefore, micro-CT could be used as a triage tool to determine which cases might be best suited for further pathological evaluation.

Whilst we focussed on image quality in this study, a recent diagnostic accuracy study showed that maceration rendered up to 50% of imaging non-diagnostic, in particular for the brain [20] and heart [9, 30], although analysis at autopsy is equally extremely challenging. Further optimisation of micro-CT may help provide greater diagnostic yield in future.

Image quality was also rated as consistently higher for head than chest imaging. This is likely due to intrinsic differences in image quality related to anatomical shapes and ensuring full inclusion within the imaging detectors: spherical objects (such as the head) can be positioned closer to the X-ray source, enabling lower EPS, whereas more elongated shapes (such as the body) require positioning further away, enforcing a higher EPS to achieve optimal coverage. This causes an increased number of projections, optimised by the scanner automatically, related to object diameter of for the head in comparison to the body, which would explain both higher image quality for the head and a negative association between increased EPS and image resolution/quality. A few fetuses which were imaged using more frames per projection chest (4/258) 2.7%, head 6/258 (2.3%), also yielded higher image quality, which is to be expected with increased signal to noise, although these scans take significantly longer, as scan time is the sum of exposure time, frames per projection and number of projections. This may explain our findings in this retrospective study, although these should be prospectively tested in a future study.

Gestational weight was negatively associated with image quality with a higher image quality rating overall for head when compared to chest images. Both these results can be explained by lower weight fetuses and smaller anatomical areas (particularly heads), resulting in a lower EPS and a higher resolution micro-CT image. Lower gestational weights and fetuses below 20 weeks gestation have significantly poorer diagnostic accuracy and image quality using other post-mortem techniques (MRI) [11] and Ultrasound [30], and should be diverted to micro-CT. Together, these factors combined influence the development of a clinical protocol for optimal resolution on a patient-by-patient basis.

We found a bimodal time-to-immersion pattern in our patient demographics, which is likely to represent differences between referral centres and their respective consent procedures. Access to the micro-CT service was expedited where full parental consent was given on referral, but delays were encountered if further information was required, results in two peaks at median 3 and 16 days (Fig. 4). This could also explain why TOP was a statistically significant positive predictor for image quality when compared to miscarriage, as these cases are likely to be referred more rapidly, also with minimal maceration. We have since adopted dedicated consent procedures at all referring centres to reduce these delays and improve both parent and professionals understanding of the procedures.

As micro-CT can generate high quality imaging in small, macerated fetuses where perinatal autopsy is known to present considerable logistical challenges, it offers an attractive alternative to those parents who do not consent to invasive autopsy and is likely to increase the uptake of post-mortem investigations [2–5]. This gives the opportunity to offer a broader range of post-mortem techniques on an individualised basis and can be useful to triage those cases in whom invasive autopsy will be of maximal yield. Formal autopsy will be of most use where post-mortem investigations and antenatal imaging findings are not concordant [31], although following fetal loss below 18 weeks gestation it is unlikely that detailed antenatal ultrasound will have been performed.

## Limitations

The main limitation was the retrospective nature of this study, where we used a relatively narrow range of imaging parameters, limited by machine capability. Were this study conducted prospectively, a wider range of imaging parameters would have been undertaken to determine optimal imaging factors, as has been performed for individual extracted organs in previous work [32]. However, this study represented “in practice” learning within our department, and we have adopted several of the key points from these results into our everyday clinical practice.

We also did not include diagnostic accuracy in this study, as several of these fetuses did not undergo comprehensive autopsy, given that our service has developed to meet a clinical demand predominantly targeted towards parents who prefer non-invasive micro-CT imaging to invasive autopsy techniques for early gestation pregnancy loss. Diagnostic accuracy has been reported elsewhere [20, 21, 25], but our results show that high image quality is possible even in severely macerated, small fetuses.

## Conclusion

High micro-CT fetal image quality is achievable following early pregnancy loss despite maceration. Higher resolution is achievable in smaller fetuses, particularly where conventional autopsy may be challenging. These factors should influence the establishing of a clinical micro-CT imaging service to address the growing need for less invasive autopsy methods.

## Data Availability

The datasets generated and/or analysed during the current study are not publicly available due to the sensitive nature of the data but are available from the corresponding author on reasonable request.

## Abbreviations

CHL: Crown-heel length
CRL: Crown-rump length
EPS: Effective pixel size
FPP: Frames per projection
HC: Head circumference
IUD: Intra-uterine death
I_2_KI: Potassium tri-iodide
kV: Kilovoltage
Micro-CT: Micro-computed tomography
PMUS: Post-mortem ultrasound
PMMRI: Post-mortem magnetic resonance imaging
TOP: Termination of pregnancy
